# High levels of tumor-associated neutrophils are associated with improved overall survival in patients with stage II colorectal cancer

**DOI:** 10.1371/journal.pone.0188799

**Published:** 2017-12-06

**Authors:** Ryan S. Berry, Meng-Jun Xiong, Alissa Greenbaum, Parisa Mortaji, Robert A. Nofchissey, Fred Schultz, Cathleen Martinez, Li Luo, Katherine T. Morris, Joshua A. Hanson

**Affiliations:** 1 Departments of Dermatology and Laboratory Medicine, Geisinger Medical Center, Danville, Pennsylvania, United States of America; 2 Department of Pathology, University of New Mexico, Albuquerque, New Mexico, United States of America; 3 Department of Surgery, University of New Mexico, Albuquerque, New Mexico, United States of America; 4 University of New Mexico School of Medicine, Albuquerque, New Mexico, United States of America; 5 Department of Surgery, University of Oklahoma, Oklahoma City, Oklahoma, United States of America; 6 Division of Epidemiology, Biostatistics, and Preventive Medicine, Department of Internal Medicine, University of New Mexico, Albuquerque, New Mexico, United States of America; National Cancer Center, JAPAN

## Abstract

Conflicting reports regarding whether high tumor-associated neutrophils (TAN) are associated with outcomes in colorectal cancer (CRC) exist. Previous investigators have counted TAN using non-neutrophil-specific immunohistochemistry (IHC) stains. We examined whether TAN levels as determined by multi-field manual counting would predict prognosis. IRB approval was obtained and two pathologists, blinded to stage/outcome, counted TAN in 20 high power fields (HPF) per specimen. TAN score was defined as the mean of these counts. High TAN was defined as at or greater than the median score for that stage. Demographics, tumor characteristics, and overall survival (OS) were obtained from the records and examined for association with TAN score. IHC for arginase expression was performed in a subset of samples. 221 patients were included. Stage II patients with high TAN scores had an OS of 232 months as compared to those with low TAN (OS = 85 months, p = 0.03). The survival benefit persisted in multivariable analysis (HR 0.48, CI 0.25–0.91, p = 0.026) controlling for age and sex. Women had increased survival as compared to men, and there were no significant prognostic associations with TAN count in stage III/IV patients, although there were only 12 stage IV patients. Arginase staining did not provide additional information. Stage II colorectal cancer patients with high TAN live nearly 3 times longer than those with low TAN. Women with stage II disease and high TAN counts appear to be driving the survival benefit seen in the stage II patients and have increased overall survival in all stages.

## Introduction

Colorectal cancer (CRC) is the second most common cancer in women worldwide, the third most common in men.[1] Approximately 75% of patients receiving adjuvant chemotherapy treatment will incur its costs and toxicities without deriving survival benefits.[2–5] New biomarkers of disease prognosis are needed.[6]

The inflammatory microenvironment of CRC is receiving more attention. New prognostic scores based on adaptive immune infiltrates are being adopted.[7] However, the role of the innate immune system within the microenvironment remains a less developed area of study. Whether there is a positive or negative prognostic effect of tumor-associated neutrophils (TAN) is not yet clear. Some studies demonstrate improved survival with higher TAN counts and others report that increasing TAN density correlates with higher stage disease and poorer survival.[8–13] These conflicting results may be due to inconsistent methods in quantifying TAN. Immunohistochemistry (IHC), with markers such as CD15 or CD66b, has been most commonly used. However, these indicators are not specific for neutrophils.[14–19] Given the fact that both eosinophil and lymphocyte infiltration have prognostic effects in CRC,[7, 20] studies using IHC with CD15 and CD66b may not detect the isolated effects of TAN alone. Finally, there is evidence suggesting that neutrophils can assume pro or anti-tumor roles in the microenvironment depending on phenotype.[21–23]

In this study, we hypothesized that TAN identified by morphology and manually counted, would provide independent prognostic information in CRC patients. Given the lack of a single immunomarker that is specific for neutrophils, we felt that a manual morphology based count would be more accurate in assessing TAN. Given that the pro-tumor neutrophil phenotype “N2” is in part defined as producing higher levels of arginase,[23] we also hypothesized that TAN with higher arginase staining would correlate with worse outcome in a subset of stage II and IV patients.

## Materials and methods

### Patient selection and follow up assessment

Following Institutional Review Board Approval and granted waiver of consent, a query of the surgical pathology database at the University of New Mexico was performed for patients with CRC who underwent resection as first course of treatment at our institution between 1991 and 2014. Patients with a history of polyposis syndromes or inflammatory bowel disease were excluded, as were patients treated with neoadjuvant therapy. Primary tumor tissue blocks were available for 246 patients. Clinical data were, however, only available for 221 of these patients. Clinical records were searched for demographics, tumor characteristics, American Joint Committee on Cancer 7^th^ edition stage (AJCC), and outcome variables. Deaths were verified by a search of the Social Security Death Index. We did not gather variables related to receipt of adjuvant chemo or radiation therapy as patients were often treated at other centers, and the records were incomplete. All patients were managed by medical oncologists who practice according to standard guidelines for therapy.

### TAN quantification

Two pathologists, blinded to patient outcomes (RSB and JAH), examined the slides and initially counted a subset of cases independently to assure minimal intra-user variability. Two representative slides were selected from each case. Ten random non-overlapping high-power fields (HPF) were then examined at 400X magnification (0.238 mm^2^) per slide (20 fields per case). TAN were defined as well preserved neutrophils within and/or immediately adjacent to neoplastic cells, including those found within areas of luminal necrosis. Areas of infarct-like necrosis and areas immediately adjacent to ulcerations were excluded. A TAN count was recorded for each field and the mean of all TAN counts per patient was recorded as the patient’s final TAN score. Given the variability of median TAN counts between stages, and the possibility that TAN infiltration may have different relationships to prognosis at different tumor stages, we defined a patient to have a high TAN count if their TAN count was equal to or above the median for that stage.

### Quantification of arginase staining and digital analysis

Staining was performed using the Ventana^™^ Discovery Platform to take slides from deparaffinization through staining. Anti-arginase antibody (Abcam ab133643) was diluted 1:250 and incubated with slides for 24 minutes at 36°. After additional standard Ventana^™^ processing and counterstaining with hematoxylin and bluing solution, slides were scanned for high-resolution digital analysis using the Aperio Scan Scope^™^ (Leica Biosystems) and digital analysis software (HALO^™^, Indica Labs). The digital analysis software was “trained” to differentiate tumor tissue, positively stained cells, and stromal tissue in an iterative approach by two pathologists (JAH and MJX). Percent neutrophils staining positive for arginase were calculated from total cells stained/total cell count. This was multiplied by the mean intensity of the staining which was scored on a scale of 0–1 to arrive at an arginase tumor score. A similar analysis was performed for the peritumoral stromal tissues to assess whether or not different locations of arginase positive neutrophils within the tumor microenvironment correlated to outcomes.

### Statistical analysis

TAN counts were classified as high if equal to or greater than the median count within that stage and low if below the median count for that stage. Overall survival was calculated from date of diagnosis to date of death from all causes or the most recent follow-up for censored patients. Kaplan-Meier method and log-rank test were used to compare estimated survival between TAN levels. Co-variates were dichotomized as follows: sex, male vs female, age, greater than/equal to 65 vs less than 65, with AJCC stage treated as an ordinal variable. All statistical tests are two-sided and variables with p<0.05 were considered statistically significant. Variables with p<0.1 were entered into the multivariable analysis using the Cox regression model. Associations of clinical and histopathological features were evaluated by Fisher’s exact test for categorical variables and Spearman’s rho for continuous variables. Statistical analysis was performed with SPSS v. 24.0 (SPSS, Chicago, IL) and SAS 0.4 (SAS, Cary, NC).

## Results

### Patient characteristics

Please see Table 1 for patient characteristics. Median follow-up was 68 months (range, 0 to 250) but 119 patients (54%) had greater than 5 years of follow-up, 77 of whom (65%) were alive at 5 years. Fifty-seven patients (26%) had a follow-up of greater than 10 years and 42 (74%) of these patients were alive at 10 years. Estimated 5- and 10-year OS rates were 80% (95% CI, 75% to 86%) and 56% (95% CI, 9% to 63%), respectively. Median survival for all stages was 68 months (range, 0 to 250). Stage-specific overall survival is summarized in Table 1.

**Table 1:** Summary of patient characteristics, n = 221.

Characteristic	n	%	Median OS (Months)	p value (comparing OS)*
Sex				
Male	107	48	74	p = 0.06
Female	114	52	108	
Age, years (Mean 61, Range 25–90)				
≥ 65 years	82	37	71	**p<0.01**
< 65 years	139	63	153	
AJCC v.7 Stage at Diagnosis				
All Stages	221	100	91	**p<0.001**
I	28	13	156	
II	89	40	133	
III	92	42	70	
IV	12	5	24	

*Comparisons of overall survival by log-rank test.

### Tumor-infiltrating neutrophils and arginase

**Fig 1:**
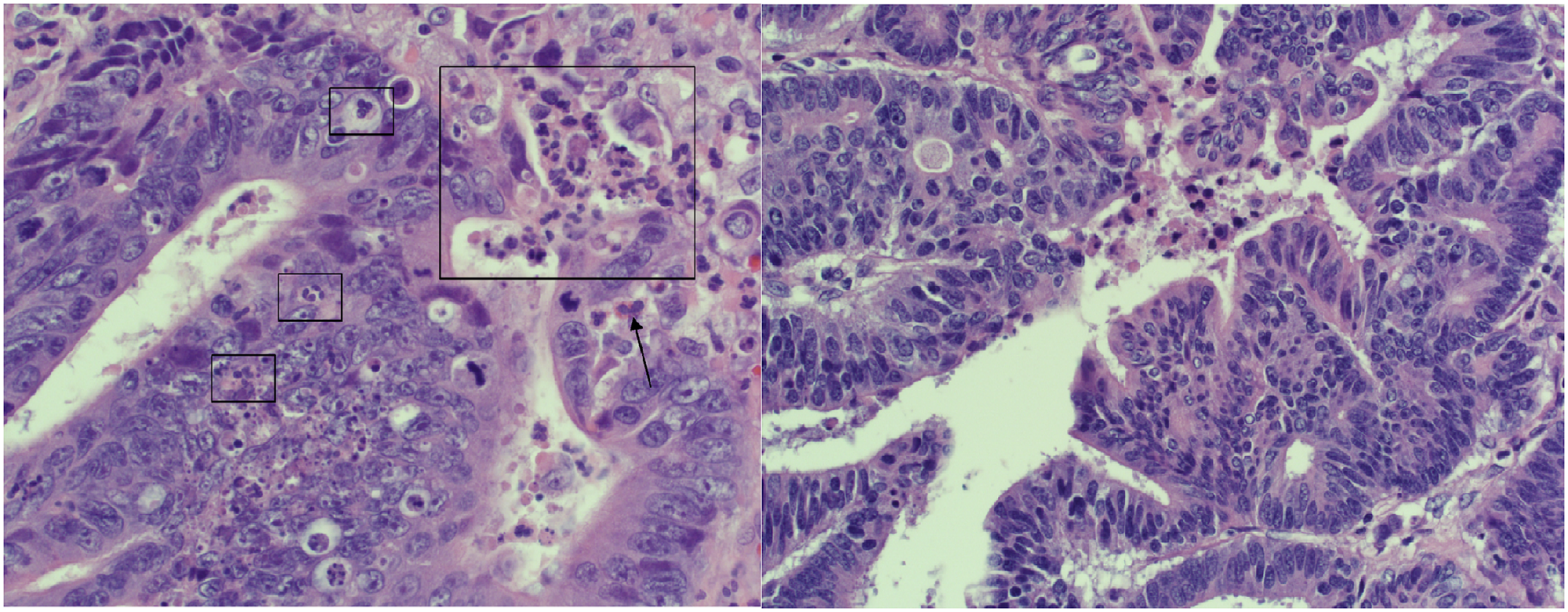
Example of low and high TAN count tumor samples. H&E of representative sections (400X) of a high TAN count tumor (left) and a low TAN count tumor (right) are shown. Examples of TAN that were counted based on morphology are shown in boxes in the high TAN count tumor, with arrows to indicate eosinophils that were not counted. There are no TANs seen in the low TAN count tumor.

**Fig 2:**
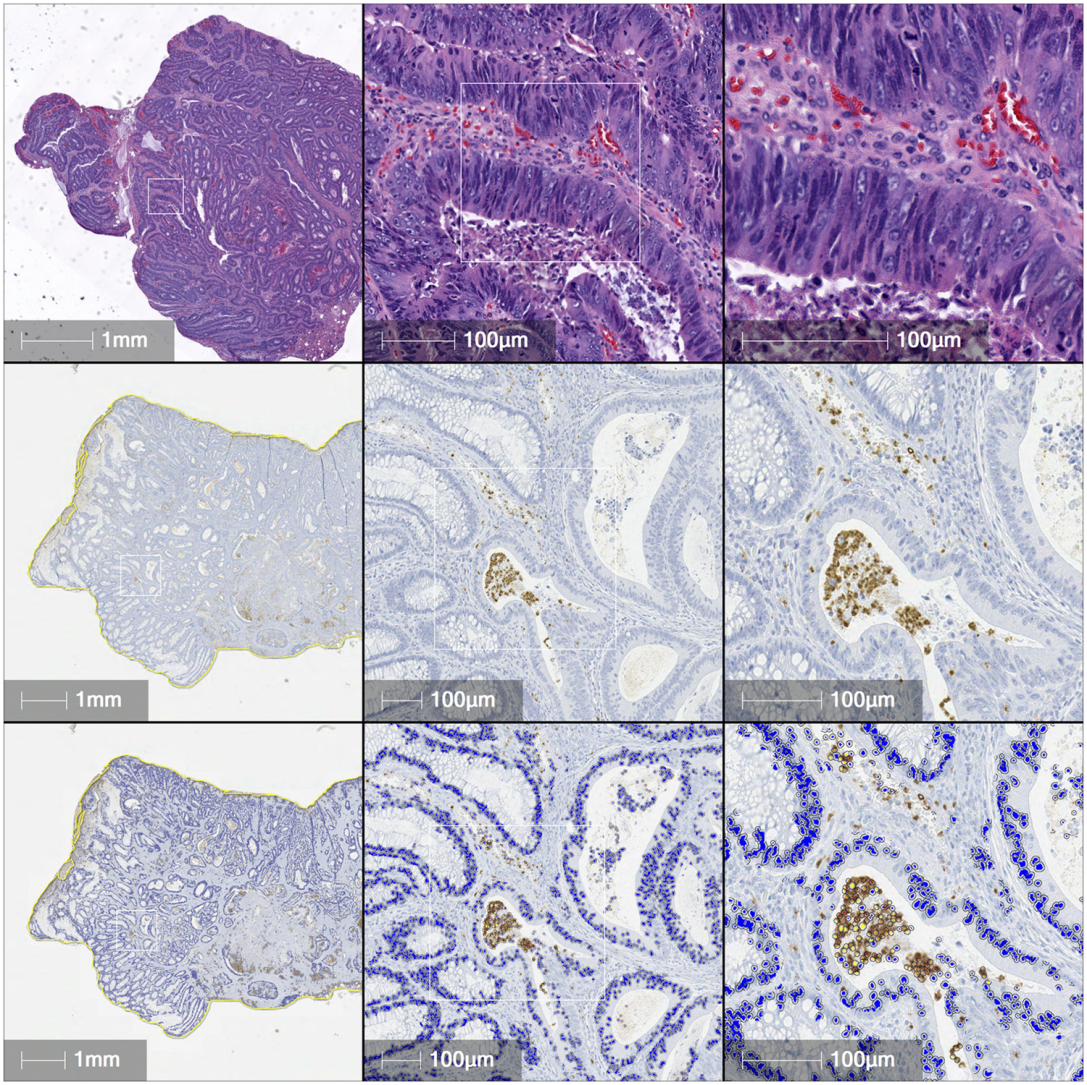
Example of staining and digital analysis. (Row A) H&E of representative section, (Row B) Arginase staining, (Row C), Halo^™^ digital analysis counting total cells (deep blue) and arginase positive cells (brown).

The median TAN count for all stages was 5.4 (range 0–140), but ranged from a median of 8.9 (range 0–39.9) in stage I patients, 4.8 (range 0.2–140) in stage II patients, 6.0 (range 0–128.6) in stage III patients, and 1.8 (range 0.2–17.2) in stage IV patients. (An example of high (left panel) and low (right panel) TAN count tumor is shown in Fig 1). The stage specific variability in the median TAN counts led us to consider whether the infiltrating cells might have different correlations to prognosis depending on tumor stage, and whether this could be due to neutrophil phenotype. Based on a readily available and optimized stain for arginase, and articles suggesting high arginase expression may be a marker of N2 or pro-tumor phenotype, [21–24] we elected to begin investigations along those lines with this stain in a subset of stage II or IV patients to determine if TAN were more likely to be of N2 phenotype in more advanced tumors. (Please see Fig 2 for examples of HPF examined, arginase staining, and digital analysis.) We found a range of arginase staining within the tumor tissue with the total median arginase score of 4.74 (range 0.072–395.2) for both stages II and IV. Stage II patients had a median score of 4.47 (range 0.072–395.2), and stage IV patients (albeit a limited sample size) had a median score of 5.64 (range 1.30–67.00), p = NS. Arginase staining within the stromal tissue was significantly higher than that within the tumor tissue (median stroma 11.26 (range 2.45–399.82) vs tumor 4.74, p<0.001), but did not differ between stage II and stage IV patients (median stage II, 11.26 vs median stage IV, 9.33, p = 0.36). TAN count and arginase tumor staining were positively correlated (Spearman’s 0.481, p<0.001) as were TAN count and arginase stromal staining (Spearman’s 0.422, p<0.001).

### Association of TAN and clinical and histopathologic variables

High levels of TAN were found to be associated with low/moderate tumor grade, though not with other clinicopathological variables (Table 2). We found a higher percentage of high grade adenocarcinomas to have low TAN, suggesting low TAN may be associated with more aggressive tumor behavior. There was no correlation between TAN level and frequency of T4 tumors or lymphovascular invasion.

**Table 2:** Comparison of clinical and pathologic features by TAN counts (n = 221).

TAN counts					
Feature	TAN low (n = 109)		TAN high (n = 112)		
	n	%	n	%	p value
Age, years					
Mean	60		62		p = 0.30
Range	25–85		37–90		
Sex					
Female	52	46%	62	54%	p = 0.28
Male	57	53%	50	47%	
Median follow up, months	70		62		p = 0.51
AJCC v.7 Stage at Diagnosis					
I	13	46%	15	54%	p = 0.19
II	44	49%	45	51%	
III	46	50%	46	50%	
IV	6	50%	6	50%	
AJCC v.7 Tumor Stage*					
T1/2	24	51%	23	49%	p = 0.87
T3/4	84	49%	88	51%	
Lymph node involvement					
Node negative	61	50%	61	50%	p = 0.89
Node positive	48	49%	51	51%	
Primary tumor site^+^					
Left colon	37	49%	38	51%	p = 0.27
Right colon	19	38%	31	62%	
Grade^					
Low/Moderate	45	42%	63	58%	**p = 0.02**
High	12	75%	4	25%	
Lymphovascular invasion (LVI)^#^					
Absent	26	44%	38	56%	p = 0.28
Present	17	53%	15	47%	

Comparisons of proportions by Fisher’s exact test. TAN = Tumor-associated neutrophils.

*Tumor stage unavailable for 2 patients, 1 in low TAN group and 1 in high TAN group.

^+^Tumor site unavailable for 96 patients, 53 in low TAN group and 43 in high TAN group. Tumor site considered left colon if primary was beyond splenic flexure.

^Grade unavailable for 97 cases, 52 in low TAN group and 45 in high TAN group.

^#^LVI incidence unavailable for 125 patients, 66 in low TAN group and 59 in high TAN group.

### Univariate survival analysis

**Fig 3:**
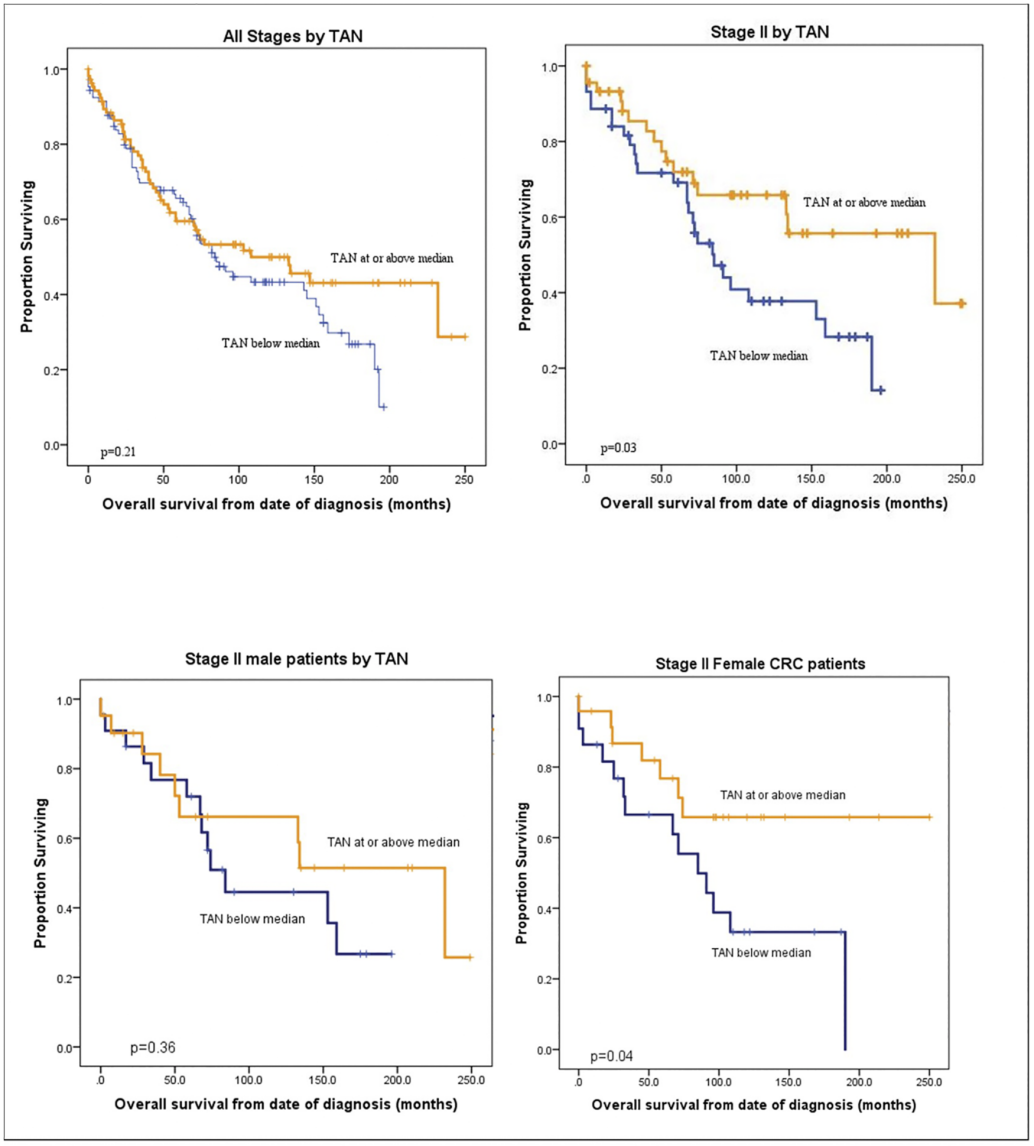
Survival curves. Overall survival probability based on High (orange) vs Low (blue) TAN **A:** All stages (n = 221, p = 0.21), **B:** stage II patients (n = 89, p = 0.03), **C: s**tage II male patients (n = 45, p = 0.36), **D:** stage II female patients (n = 46, p = 0.04) TAN = Tumor-associated neutrophils.

High TAN counts predicted overall survival only in patients with stage II disease on univariate analysis. Among patients with stage II CRC, high TAN counts were significantly associated with a nearly *3-fold* increase in overall survival compared to those with low TAN counts (Table 3, Fig 3B). In addition, in women there was a trend towards better overall survival in all patients with high TAN counts (high TAN overall survival 216 months vs low TAN overall survival of 87 months, p = 0.116) suggesting a potential for different roles for TAN in men and women with CRC. Furthermore, the survival benefit seen in stage II patients with high TAN appeared to be driven by the women (Fig 3C and 3D).

**Table 3:** The association between TAN categories and overall survival within strata using univariate analysis (n = 221).

Variable	n (%)	Median TAN	Median OS (Months)	Median OS High TAN (Months)	Median OS Low TAN (Months)	p value
Stage						
I	28 (13%)	8.85	156	216	156	p = 0.59
II	89 (40%)	4.8	133	232	85	**p = 0.03**
III	92 (42%)	6.0	70	70	71	p = 0.64
IV IV	12 (5%)	1.8	24	24	24	p = 0.56
All Stages	221 (100%)	5.4	91	133	82	p = 0.12
Sex						
Male (All stages)	107	4.3	74	77	74	p = 0.71
Female (All stages)	114	7.0	108	216	87	p = 0.12
Male (Stage II)	43	4.3	134	232	84	p = 0.36
Female (Stage II)	46	5.1	108	Not reached	85	p = 0.04

TAN = Tumor-associated neutrophils.

### Multivariable analysis

We found a marginal protective effect for female sex and younger age in initial univariate analysis (Table 1). These variables, in addition to AJCC stage, were included in multivariable analysis using Cox regression models. In Stage II patients adjusted for age and sex, a high TAN count demonstrated a protective effect that was significant (HR 0.48, 95% CI 0.25–0.91, p = 0.026). This protective effect was not found when all stages were considered together on multivariable analysis.

## Discussion

Tumor-associated macrophages have been shown to be an important stage specific prognostic predictor in CRC but there is conflicting evidence regarding the prognostic impact of TAN.[24] To address the variation in methodology and reliance on non-neutrophil-specific antibodies in assessing TAN counts in the literature, as well as emerging data regarding differing neutrophil phenotypes, we sought to evaluate the prognostic significance of TAN as assessed by morphologic inspection and limited phenotyping as morphology is the most reliable means to distinguish neutrophils from other inflammatory cells. To our knowledge, this is the first study reporting results in CRC when TAN are measured in such manner. We found a prognostic effect for patients with stage II disease, as patients with tumors high in TAN demonstrated a 3-fold OS increase compared to those with low TAN counts. In addition, Stage IV tumors had the lowest median TAN count. Arginase staining did not add any prognostic information to manual TAN counts. Interestingly, we also found a trend towards women having better outcomes than men, with a larger difference in OS based on high vs low TAN counts, suggesting a unique role for the innate immune system in sex-based survival differences for this tumor. Given the challenges in predicting which stage II CRC patients will most benefit from chemotherapy, it is possible that TAN count could be used to identify patients with a worse prognosis who might benefit from adjuvant therapy.

Previous studies have reached differing conclusions regarding the effect of TAN in cancer survival and limited papers have explored the role of TAN in CRC specifically. Rao et al found high intratumoral neutrophil counts, defined as >60 CD66b+ cells per tumor microarray (TMA) spot, to be associated with worse survival.[11] However, as previously mentioned, the CD66b marker is not specific for neutrophils and the use of TMA fails to account for tumor heterogeneity in the same way that our method of scoring 20 separate HPFs on whole sections does. In addition, those investigators defined high TAN counts using a single number derived from receiver operating characteristic curve analysis for all stages based on best sensitivity and specificity for predicting metastatic status. Given that we found such variation in TAN count medians between stages, we elected to define high TAN by the stage specific median, which may have given differing results.

Our results are consistent with studies by Galdiero et al. and Wikberg et al showing improved prognosis with higher TAN in CRC patients.[8, 25] We observed similarly different median TAN counts by stage, as well as the lowest TAN density in stage IV tumors. Galdiero et al used disease-specific and disease-free survival as their endpoints and found that the positive prognostic effect for high TAN counts extended to patients with all stages of disease. Both groups used CD66b+ to define neutrophils, however, thereby possibly confounding the results with inadvertent counting of eosinophils. Our results suggesting benefits for high TAN only in stage II patients suggests a stage specific role for TAN, which fits with evidence for a stage specific prognostic association for tumor-associated macrophages as well in CRC.[24] Intriguingly, Galdiero et al. also demonstrated improved sensitivity to 5-FU treatments in their stage III patients who had tumors with high TAN. Given that the stage II patients with low TAN tumors in our series had an OS closer to that of stage III patients, we feel they should also be considered for adjuvant chemotherapy, while acknowledging that the Galdiero paper suggests they may have less benefit from 5-FU alone. The effects of TAN count on predicting response to modern regimens such as FOLFOX and FOLFIRI are unknown, however, and warrant further study.

Our study unsuccessfully attempted to gain more prognostic information from a preliminary attempt to phenotype the neutrophils with arginase staining. However, we had an extremely limited number of primary tumors from stage IV patients to evaluate. Therefore, additional measurement of TAN and arginase staining in stage IV patients should be performed because we do not feel our study sufficiently evaluated the possibility for manual TAN counts to have prognostic value in stage IV patients. If arginase did indeed correlate with an “N2” phenotype, it is reasonable to hypothesize that increased arginase staining in TAN would be associated with worse outcomes in stage IV patients. Finally, given the use of digital analysis to quantify arginase staining, it is possible that we captured both positively staining neutrophils and positively staining macrophages as macrophages also express arginase.[26] Future work in this area will require careful assessment of the cell type that is staining for arginase. For now, all that can be concluded from this work is that there is a higher level of arginase staining in the CRC stroma, and that TAN counts and arginase staining of the total tumor tissue were positively correlated.

Our study includes a few limitations. It is retrospective in nature, thereby limiting our ability to control for selection bias or misclassification. However, patient information is collected in the tumor registry prospectively. We could not ascertain the receipt and type of chemotherapy which may affect survival outcomes, and we do not have data regarding microsatellite instability (MSI). MSI is a powerful prognostic indicator in CRC but the beneficial effects are believed to be mediated through the adaptive immune response. TAN, on the other hand, as a component of the innate immune system, are unlikely to be correlated with MSI status. However, given financial limitations, we were not able to specifically test for a potential association and therefore view this as a minor limitation.

In summary, we employed a morphology based method to manually measure TAN counts in CRC patient samples. We found higher TAN counts in Stage II patients are associated with a nearly 3-fold increase in OS compared to patients with low TAN. TAN counts may provide a means to further identify stage II patients with a worse prognosis so they could be considered for adjuvant chemotherapy.

## Supporting information

S1 File 2017 Morris Plos One TAN and CRC. (XLSX)
